# Postnatal and Adult Aortic Heart Valves Have Distinctive Transcriptional Profiles Associated With Valve Tissue Growth and Maintenance Respectively

**DOI:** 10.3389/fcvm.2018.00030

**Published:** 2018-04-24

**Authors:** Emily Nordquist, Stephanie LaHaye, Casey Nagel, Joy Lincoln

**Affiliations:** ^1^Molecular Cellular and Developmental Biology Graduate Program, The Ohio State University, Columbus, OH, United States; ^2^Center for Cardiovascular Research, The Research Institute at Nationwide Children's Hospital, Columbus, OH, United States; ^3^The Heart Center, Nationwide Children’s Hospital, Columbus, OH, United States; ^4^The Institute for Genomic Medicine at Nationwide Children’s Hospital, Columbus, OH, United States; ^5^Ocean Ridge Biosciences, Deerfield Beach, FL, United States; ^6^Department of Pediatrics, The Ohio State University, Columbus, OH, United States

**Keywords:** aortic valve, RNA-sequencing, mRNA, cell proliferation, extracellular matrix, postnatal, adult

## Abstract

Heart valves are organized connective tissues of high mechanical demand. They open and close over 100,000 times a day to preserve unidirectional blood flow by maintaining structure-function relationships throughout life. In affected individuals, structural failure compromises function and often leads to regurgitant blood flow and progressive heart failure. This is most common in degenerative valve disease due to age-related wear and tear, or congenital malformations. At present, the only effective treatment of valve disease is surgical repair or replacement and this is often impermanent and requires anti-coagulation therapy throughout life. Therefore, there is a critical need to discover new alternatives. A promising therapeutic area is tissue regeneration and in non-valvular tissues this requires a tightly regulated genetic “growth program” involving cell proliferation. To explore this in heart valves, we performed RNA-seq analysis to compare transcriptional profiles of aortic valve tissue isolated from mice during stages of growth (postnatal day (PND) 2) and adult maintenance (4 months). Data analysis reveals distinct mRNA profiles at each time point and pathway ontology identifies associated changes in biological functions. The PND2 aortic valve is characterized by extensive cell proliferation and expression of mRNAs related to the extracellular matrix (ECM). At 4 months, proliferation is not significant and a differential set of ECM-related genes are expressed. Interestingly there is enrichment of the defense response biological process at this later time point. Together, these data highlight the unique transcriptome of the postnatal valve during stages of growth and maturation, as well as biological functions associated with adult homeostatic valves. These studies create a platform for future work exploring the molecular programs altered in the onset of heart valve disease after birth and provide insights for the development of mechanistic-based therapies.

## Introduction

The average heart beats over a billion times during one lifespan to continuously provide blood to every part of the body. Crucial to this task are the four heart valves (aortic, pulmonic, tricuspid and mitral) that function to maintain the unidirectional blood flow. Distinct from the cardiac muscle, the mature valve leaflets are highly organized structures comprised of three layers of extracellular matrix (ECM) components including collagens, proteoglycans, and elastin (1). Formation and maintenance of the valve ECM is mediated by a heterogeneous population of valve interstitial cells (VICs) that are fibroblast-like in phenotype (2). Surrounding the VICs and ECM is a single layer of valve endothelial cells (VECs) that physically protect the valve from external stimuli, and molecularly communicate with underlying VICs to regulate homeostasis of the ECM (3–5). The complex relationship between valve cell populations and the ECM is critical for establishing and maintaining structure-function relationships throughout life. This relationship begins during embryonic development, as mesenchymal precursor cells in the endocardial cushions transition towards an activated VIC phenotype and degrade primitive ECM within the cushions while secreting more diverse ECM components. Elongation and remodeling of the immature valve structures continues for a short time during the postnatal period until around day 10 in the mouse when the ECM components are more defined. Once valve formation is complete, VICs convert to a quiescent phenotype and in the absence of disease, maintain physiological turnover of the ECM to provide efficient function throughout life [reviewed (2, 6)]. While the regulation of valve development is well established, the mechanisms that regulate postnatal valve growth and remodeling, as well as adult homeostasis are poorly understood. Despite constant mechanical demand on the valve leaflets, turnover of valve cell populations in adult valves is relatively low (4). Therefore, it remains unclear how valve cell populations and structure-function relationships are maintained throughout life in healthy individuals, yet dysregulation of these relationships likely underlie the onset and progression of valve dysfunction and disease.

Heart valve disease is a growing public health problem that can affect both adult and pediatric patients. Significant defects during embryonic valve development lead to congenital malformations which compromise the typical structure of the valve, often resulting in reduced ability to function correctly [reviewed (7)]. Distinct from valve disease present at birth, pathology can also be acquired and is most prevalent in the aging population, with up to 13% of people aged over 75 affected by diseases including calcification or myxomatous degeneration (8). Currently, the only effective treatment for valve disease is surgical repair or replacement, resulting in over 90,000 valve replacement surgeries performed in the US each year (9). Surgical treatment comes with many complications including the need for repeat surgeries due to low valve durability and high thrombogenicity, in addition to the large personal and societal economic burdens (10). Therefore, there is a critical need for the development of alternate therapeutics.

A promising therapeutic area is emerging in the field of self-repair and regenerative medicine. Common to both congenital and age-related valve disease is the damage and consequent loss of healthy cell populations alongside the development of pathological cell populations that are therefore unable to preserve valve structure-function relationships (11). The field of cardiac regeneration has recently made significant advances in elucidating the molecular mechanisms of regeneration, and it has been reported that the neonatal myocardium has remarkable regenerative capacity during the first seven days of life (12–14). Furthermore, several pathways have been identified as key players, and the ability to recapitulate these neonatal programs in adults has proven successful in promoting myocardial regeneration after injury and in disease models (15–18).

Neonatal, adult and potential regenerative programs have not been examined in heart valves and therefore the goal of this current study is to initiate this discovery. To do this, we used RNA-seq analysis to explore differential molecular profiles between postnatal and adult valve cell populations. This analysis will help define potential regeneration indicators that in the future might be reintroduced in diseased or aging adult valves to increase their self-repair capacity and improve structure-function relationships. Our study has defined transcriptional differences between postnatal day 2 (PND2) and adult (4 months) aortic valves and identified significant changes in key biological functions related to cell proliferation, ECM, and defense response that may be important for determining the regenerative capacity of the valve to aid in the future development of alternative therapeutics.

## Material and Methods

### Mice

*C57BL/6J* mice were fed regular chow mix and housed in a controlled environment with 12 h light/dark cycles at 21°C and 23% humidity and water ad libitum. Animals were euthanized by CO_2_ exposure followed by secondary euthanasia by cervical dislocation (adult mice) or decapitation (pups). All animal procedures were approved by The Research Institute at Nationwide Children’s Hospital Institutional Animal Care and Use Committee (Protocol # AR13-00054).

### Tissue Preparation

Hearts were collected from postnatal day 2 (PND2) and 4 month old *C57BL/6J* mice and fixed in 4% paraformaldehyde/1xPBS overnight at 4°C. For paraffin sections, tissue was embedded in paraffin wax and sectioned at 10 µm. Paraffin was removed in xylene, and tissue sections were re-hydrated through a graded ethanol series and rinsed in 1xPBS as previously described (19). Tissue sections containing aortic valves were then subjected to Movat’s Pentachrome staining, EdU staining, or immunohistochemistry/immunofluorescence (described below). For cryo sections, tissue was embedded in OCT and frozen, then sectioned at 7 µm. Prior to staining, tissue was permeabilized using 0.1% Triton-X 100 in 1xPBS and then subjected to immunofluorescence staining.

### Immunohistochemistry/Immunofluorescence

Whole hearts from PND2 and 4 month old *C57BL/6J* mice were collected and prepared according to above methods. Movat’s Pentachrome staining was performed on paraffin tissue sections at each time point according to the manufacturer’s instructions (Russel Movat, American MasterTech, #KTRMP), then mounted using VectaMount Permanent Mounting Medium (Vector Laboratories, H-5000). For antibody detection, fixed paraffin tissue sections were subjected to antigen retrieval by boiling for 10 min in unmasking solution (Vector Laboratories), and both cryo and paraffin sections were subjected to blocking for 1 h at room temperature (1% BSA, 1% cold water fish skin gelatin, 0.1% Tween-20/PBS) as described (20). Tissue sections were then incubated overnight at 4°C or 1 h at room temperature with primary antibodies against Mmp3 (rabbit, 1:100 paraffin, Abcam ab53015), Nid2 (rabbit, 1:200 cryo, Abcam ab14513), Ptgs2 (Rabbit, 1:100 paraffin, Cell Signaling 12282), and Rarres2 (Mouse, 1:100 paraffin, Santa Cruz sc-373797). For immunofluorescent primary antibody detection of Mmp3, Nid2, and Ptgs2, sections were incubated for 1 h at room temperature with Donkey anti-rabbit or Goat anti-rabbit Alexa-Fluor IgG secondary antibodies (1:500) (LifeTechnologies), then mounted in Vectashield anti-fade medium with DAPI (Vector Laboratories) to detect cell nuclei. For diaminobenzidine (DAB) staining of Rarres2, sections were stained using Mouse and Rabbit Specific HRP/DAB (ABC) Detection IHC kit (Abcam, ab64264), counterstained with hematoxylin (Vector Laboratories, H-3404), and mounted using VectaMount Permanent mounting medium (Vector Laboratories, H-5000). Images were visualized using an Olympus BX51 microscope and captured using an Olympus DP71 camera and CellSens software. Image brightness and contrast were edited using Adobe Photoshop CC.

### EdU Staining and Quantification

PND2 and 4 month old *C57BL/6J* mice were injected subcutaneously with 10 µg/g body weight EdU (Invitrogen) dissolved in 1xPBS. 24 h later, mice were sacrificed and hearts were collected and prepared according to above methods. Fixed tissue sections were blocked for 1 h at room temperature (1% BSA, 0.1% Cold water fish skin gelatin, 0.1% Tween 20 in PBS with 0.05% NaN_3_), followed by use of Click-it EdU Kit (Invitrogen) to detect presence of EdU according to the manufacturer’s instructions. Sections were then mounted in Vectashield anti-fade medium with DAPI (Vector Laboratories) to detect cell nuclei. The total number of cell nuclei in one leaflet were counted using ImageJ cell counter. The number of EdU + cells were then counted and calculated as a percentage of total cells. An average of 9 leaflets were counted and averaged for each mouse, with a total *n* = 3. Statistical analysis was performed in GraphPad Prism 7.0a.

### Aortic Valve Isolation and RNA-Sequencing

Aortic valves from wild type PND2 and 4 month old *C57BL/6J* mice were isolated with minimal myocardial contamination and immediately flash frozen in liquid nitrogen. Frozen samples were sent to Ocean Ridge Biosciences LLC (Deerfield Beach, FL), where RNA isolation and sequencing was performed as follows. Total RNA was extracted using the TRI Reagent® (Molecular Research Center; Part #: TR118) method, and isolated RNA was quantified using chip-based capillary electrophoresis (Agilent 2100 Bioanalyzer Pico Chip). RNA was digested with RNase free DNase I (Epicentre; Part # D9905K) and purified through minElute columns (Qiagen; Part #: 74204). Final RNA samples were quantified by O.D. measurement and re-quantified using chip-based capillary electrophoresis. Amplified cDNA libraries were prepared from 200 ng on DNA-free total RNA using TruSeq Stranded Total mRNA Library Prep Kit LT (Illumina Inc.; Part #s: RS-122-2101 and RS-122-2102). Chip-based capillary electrophoresis was used to assess quality and size distribution of the libraries. KAPA Library Quantification Kit (Kapa Biosystems, Boston, MA) was used to quantify the libraries. Libraries were pooled at equimolar concentrations and were clustered on an illumina cBot cluster station. Clustering was performed with the HiSeq PE cluster kit v4 and sequenced on an Illumina HiSeq Flow Cell v4 with 50 nt paired-end reads plus dual index reads using the Illumina HiSeq SBS Kit v4. An average of approximately 48.3 million passed-filter 50 nucleotide paired-end reads were obtained per sample (24.1M per direction).

Raw FASTQs were split into files containing 4,000,000 reads and checked for quality using the FASTX-Toolkit. The reads were filtered (removing sequences that did not pass Illumina’s quality filter) and trimmed based on the quality results (3 nucleotides at the left end of the R1 reads and 1 nt at the left end of the R2 reads). Sequence alignment was performed using TopHat v2.1.0 to the mm10 genome. BAM files were merged on a per sample. Exon and gene level counting were performed using the easyRNASeq version 2.4.7 package. A binary annotation file, built using the annotation file generation function of EasyRNASeq, was used for this analysis; the Ensembl release 83 GTF file was used as input. Annotation was performed using a Gene Transfer Format (GTF) annotation file for Mus musculus, which was downloaded on February 11, 2016 and contains the current Ensembl Mouse release 83. Filtering of the RPKM values was performed to retain a list of genes with a minimum of approximately 50 mapped reads in 25% or more samples. The threshold of 50 mapped reads is considered the Reliable Quantification Threshold, as the RPKM values for a gene represented by 50 reads should be reproducible in technical replicates. To avoid reporting large fold changes due to random variation of counts from low abundance mRNA, RPKM values equivalent to a count of ≤10 reads per gene were replaced with the average RPKM value equivalent to 10 reads/gene across all the samples in the experiment.

An unpaired two-sample heteroscedastic *t*-test was performed on the log_2_ RPKM values to compare the overall effects of age (PND2 or 4 month) on gene expression. Fold changes were also calculated for 4 month / PND2 using the mean of each group being compared. If the mean of both groups considered in the fold change comparison was below RQT, “NA” is reported. All statistical analysis was performed using R version 3.2.2 statistical computing software. A total of 7,496 genes were determined to have a low FDR-value (FDR <0.1) for the unpaired *t*-test. Full dataset is available through NCBI GeoDatasets, accession code GSE108083, “RNA-seq analysis of aortic heart valves in mice”.

### RNA-Sequencing Data Analysis

A heatmap was generated from 23,303 differentially expressed genes. Log2 transformed RPKM values were utilized and hierarchical clustering analysis was performed with Cluster 3.0 software (21). Genes and samples were clustered using centered correlation as the similarity measure and average linkage as the clustering method. A volcano plot was generated utilizing ggplot2 and is plotted as the -Log10(*p*-value) vs. Log2 Fold Change. The volcano plot highlights the differential gene expression between postnatal day 2 and 4 month aortic valves. A Venn diagram was generated based only on genes with a low *t*-test *p* value (*p* < 0.05), a fold change >2, and RPKM values above the Reliable Quantification Threshold for all biological replicate samples from either group. If at least one of the gene reads from a triplicate set was proven undetectable while all gene reads in the comparative sample set was proven detectable, the gene was considered to be uniquely expressed. If the gene read from both triplicate sample sets had detectable RPKM values about the Detection Threshold, the gene was considered common amongst sample groups. Genes with at least one triplicate below the Detection Threshold in both samples sets are not represented in the Venn diagram.

Functional annotation was performed through the utilization of Database for Annotation, Visualization, and Integrated Discovery (DAVID) version 6.8 (22). 2,082 differentially expressed genes with an FDR <0.05 and fold change >2, were assessed utilizing Gene Ontology (GO) FAT terms, which were employed to filter out broad GO categories based on a measured specificity of each term. Visualization of GO term analysis was performed using the GOPlot R package version 1.0.2 (23). To reduce the redundancy of GO terms, the reduce overlap function was used, with the threshold set to 0.75, which removes GO terms that have a gene overlap greater than or equal to the set threshold. Bubble plots were generated for the reduced GO term list using the GoBubble function, and the top 15 GO terms from biological processes, cellular component, and molecular function were visualized. Each bubble represents a term, where the size of the bubble correlates to the number of genes within the term, and it is plotted as –log (FDR) vs. z-score. The z-score is a crude measurement, predicting if a term will be upregulated or downregulated, and is calculated by taking the number of upregulated genes and subtracting the number of downregulated genes and then dividing this number by the square root of the number of genes in each pathway. The circle plot was generated using the GoCircle function, and highlights the gene expression changes within each of the selected terms. The circle plot highlights the overall gene expression change by showing increased expression in red and decreased expression in blue. The circle plot also highlights the *p*-value of the GO term by the height of the inner rectangle, which is also colored by z-score. A chord plot was generated using the GoChord function, and it represents 59 differentially expressed genes and their correlation to the following associated terms: extracellular matrix, cell proliferation, cell cycle, mitotic cell cycle process, defense response, and regulation of immune system processes. The chord plot also highlights the log fold change of each differentially expressed gene that is shown. 

### qRT-PCR

RNA was extracted from isolated aortic valves from PND2 and 4 month old *C57BL/6J* mice to validate RNA-seq findings. Briefly, Trizol reagent (Invitrogen) was used to extract RNA according to manufacturer’s instructions, and cDNA and PCR reactions were performed as previously described by our lab (24). Primers for genes selected for validation were designed in NCBI Primer-BLAST based on FASTA sequence and shown below:

**Table:** 

**Gene name**	**Forward primer sequence (5^**′**^ to 3^**′**^)**	**Reverse primer sequence (5^**′**^ to 3^**′**^)**
***Nid2***	AGGAGTGAGCATGTTTCGG	AGGGGTATTGCCAGCTTCAC
***Mmp3***	TGCATGACAGTGCAAGGGAT	ACACCACACCTGGGCTTATG
***Marckls1***	CCCGTGAACGGAACAGATGA	CCCACCCTCCTTCCGATTTC
***Gsn***	GGGACGGCCGGTTACTTAAA	CTTCAGGAATTCGGGGTGCT
***Filip1l***	AGGCTCCACTGCTGGATTTC	GACTTCTCTGACACGGGACG
***Myoc***	ACGACACTAAAACGGGGACC	TTCTGGCCTTTGCTGGTAGG
***Retnla***	GGAACTTCTTGCCAATCCAGC	CAGTGGTCCAGTCAACGAGT
***Npdc***	GCACTCCCGACACTTTTCTC	GGTACCCACTCCGGGAACT
***Sfrp4***	CCTGGCAACATACCTGAGCA	AGCATCATCCTTGAACGCCA
***Mki67***	AGAGCTAACTTGCGCTGACT	ACTCCTTCCAAACAGGCAGG
***Nrg1***	CCATCTCTCGATGGGCTTCC	ATGCAGAGGCAGAGGCTTAC
***Nrep***	GCATGATGCCCTTTTTCATCCA	TCCTTAGGCACGGGAAGTCT
***Acta2***	CCTTCGTGACTACTGCCGAG	GAAGGTAGACAGCGAAGCCA
***Dlk1***	AGAGTACCCCTCTCCTCACC	CGCCGCTGTTATACTGCAAC
***Cfd***	TACATGGCTTCCGTGCAAGT	GGGTGAGGCACTACACTCTG

Quantitative real-time PCR using a Step One Plus Real Time PCR system (Applied Biosystems) was used to detect changes in gene expression with Sybr Green reagents. Cycle counts for each target gene were normalized to *β-actin* expression and differences in gene expression were reported as a fold change from the 4 month time point. Statistical analysis was performed in GraphPad Prism 7.0a.

## Results

### Postnatal Valve Maturation and Adult Maintenance Are Associated with Distinct Transcriptional Profiles

**Figure 1:**
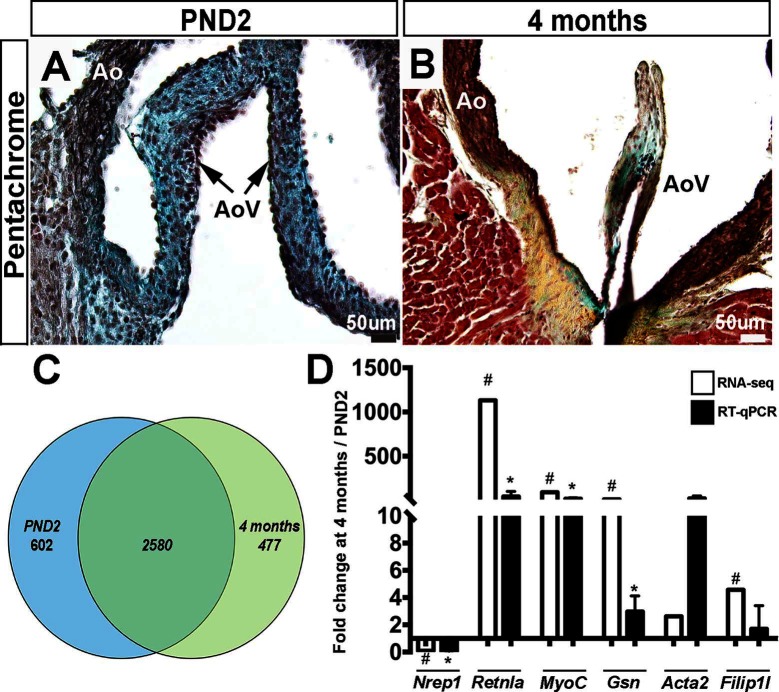
Postnatal and adult aortic vales display differences in ECM composition and gene expression. Movat’s Pentachrome staining to show extracellular matrix composition at PND2 **(A)** and 4 months **(B)** (black: elastic fibers and cell nuclei, blue: proteoglycans, yellow: collagen, red: muscle and fibrinoids). **(C)** Venn diagram to show distribution of the detected mRNAs that were uniquely or commonly expressed at PND2 or 4 months. **(D)** RT-qPCR validation (black bars) of RNA-seq findings (white bars) (*n* = 3, *:*p* < 0.05; two-tailed unpaired *t*-test, #:FDR <0.05). Ao, aorta; AoV, aortic valve.

As previously described, the valve structures continue to grow and remodel after birth (1). As shown by Movat’s Pentachrome stain, murine aortic valve structures at postnatal day 2 (PND2) are thick and composed of predominantly proteoglycan (blue), with less extensive collagen and elastin (Figure 1A). By 4 months of age, the leaflets have elongated and display distinct layers of collagen (fibrosa, yellow), proteoglycan (blue), and elastin (black) (Figure 1B).

**Figure 2:**
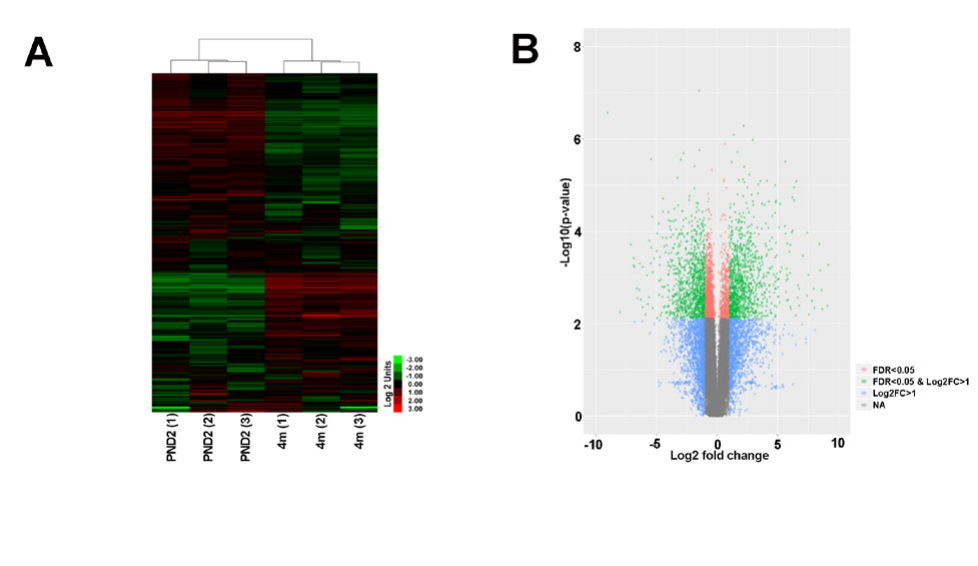
RNA-seq analysis reveals transcriptional differences between PND2 and 4 month old murine aortic valves. **(A)** Hierarchical heatmap cluster shows clustering analysis of biological replicates (*n* = 3). **(B)** Volcano plot of differentially expressed genes sorted according to fold change and significance (FDR). Green dots represent genes with log2 fold change >1 and FDR <0.05, pink dots represent genes with FDR < 0.05, and blue dots represent genes with log2 fold change >1.

In order to further define molecular profiles associated with the structural changes in postnatal and 4 month old aortic valves, we performed RNA-sequencing on isolated samples. Overall, RNA expression for samples consistently clustered by time point, as shown several ways including a Pearson’s correlation matrix (data not shown), principal component analysis (PCA) (data not shown) and hierarchical heatmap (Figure 2A). Of 23,303 detectable genes, 3,659 genes were found to have a *p*-value < 0.05 and a fold change >2, and include 1,858 upregulated and 1,801 downregulated transcripts. Of these 3,659 differentially expressed genes, 602 were unique to the PND2 time point and include *Dlk1*, *Hif3a, Agtr2* and *S100A9*, while 477 were only expressed at 4 months (*Cfd, Rtn1a, Clec3a, Adipoq, etc.*), leaving 2,580 common to both groups (Figure 1C). Table 1 includes the top 20 mRNAs uniquely expressed at each time point based on RPKM value, which is indicative of mRNA abundance. Additional RT-qPCR analysis of independent cDNA samples validated RNA-seq findings in 10 out of 12 genes (85%) at a significance threshold of *p* < 0.05 (Figures 1D, 4A and 5A,D and data not shown).

**Table 1:** Top 20 unique genes at PND2 and 4 month time points.

**PND2**	**4** Month
Dlk1	Cfd
Hif3a	2210407C18Rik
Agtr2	Retnla
S100a9	Clec3a
Slc38a5	Adipoq
Col24a1	Ces1d
Bmp7	Thrsp
Vash2	Pck1
Igf2bp3	Mgl2
Stfa1	C7
S100a8	Inmt
Cited1	Cidec
Gm5483	Fmo3
Frem2	Angpt4
Gipr	Hamp
Ube2c	Art1
Dctd	Tmem45b
1110032F04Rik	Olfr224
C1qtnf3	Plin1
Cdkn3	Rpl3l

Genes are listed in order of RPKM with the most highly expressed at the top of the list. Only genes with a low t-test p value (t-test P: Age < 0.05), a Fold Change: 4mo/P2 >2 up/down, and above the Reliable Quantification Threshold in all samples from either group (i.e., the gene RPKM values were >RQT in all three P2 samples or in all three 4mo samples) were retained for further filtering.

### Transcriptional Analysis Identifies Age-Dependent Transcriptional Profiles and Biological Functions

**Figure 3:**
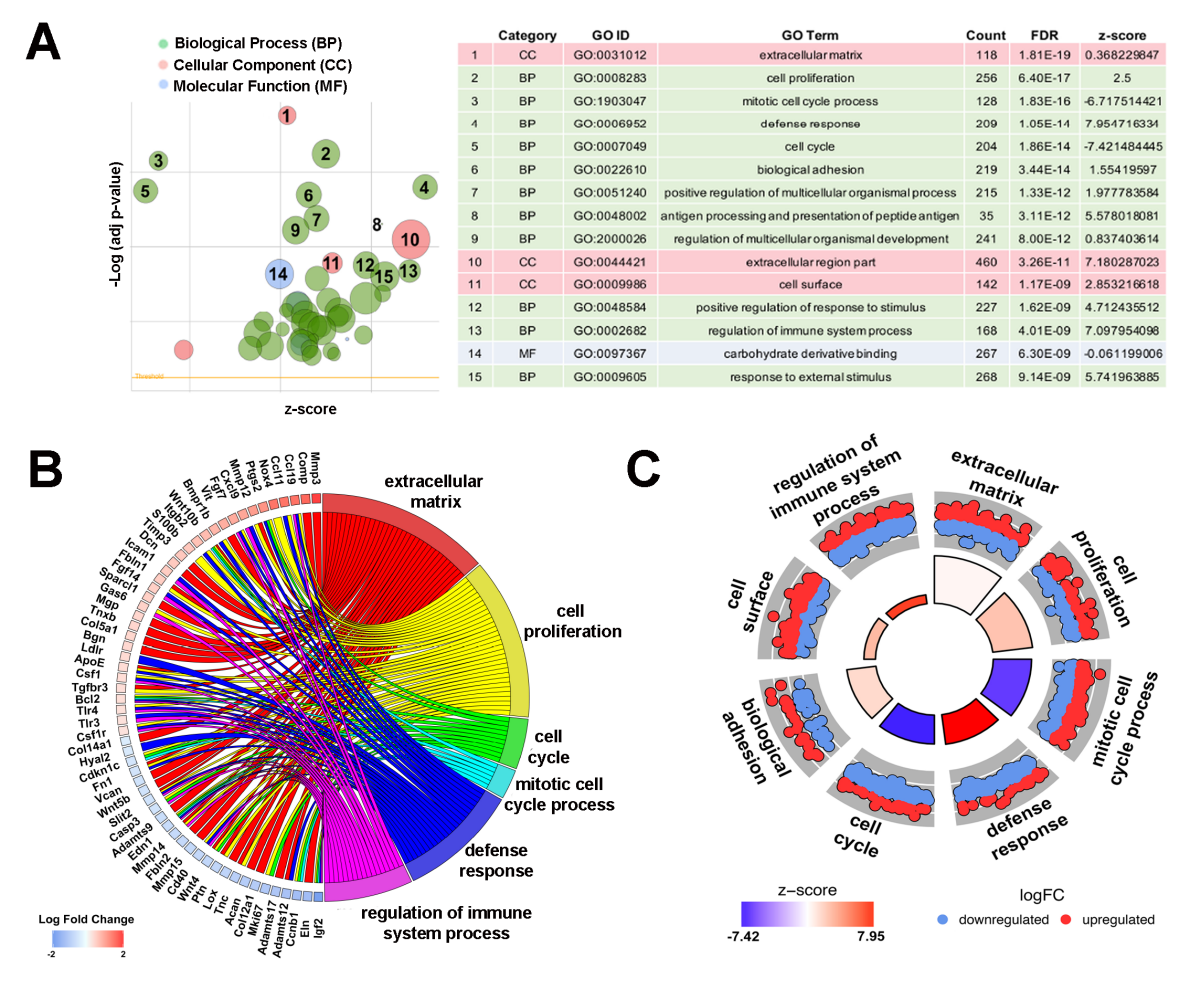
Pathway analysis identifies diverse key biological processes, cellular components and molecular functions between PND2 and 4 month old murine aortic valves. **(A)** Gene ontology (GO) analysis reveals top differentially regulated GO terms, graphically displayed according to significance (*p*-value) and z-score, a measure of overall up or down regulation for the category. Data represented as a bubble plot (left). The size of each circle represents the number of differentially expressed genes in that GO term, while color represents the category (green: biological process pink: cellular component, blue: molecular function). Table (right) lists the top GO terms along with count, FDR, and z-score. **(B)** Chord plot showing 59 differentially expressed genes previously associated with healthy and diseased valves, and their overlap between significant GO terms as determined in **(A)**. Color below each gene corresponds to log fold change, with red representing increased expression at the 4 month time point and blue representing decreased expression at 4 months. **(C)** Circular plot highlighting gene expression differences within each selected GO term, with each red dot depicting a gene upregulated at 4 months in that category, and each blue dot showing a gene downregulated at 4 months. The height of the inner rectangle represents the *p*-value of the GO term and is colored according to z-score, with red being increased at the 4 month time point and blue being decreased at 4 months.

Heatmap hierarchical clustering analysis, where 23,300 differentially expressed genes and samples were clustered using center correlation as the similarity measurement and average linkage as the clustering method, revealed molecular similarities between biological replicates at each time point and distinct differences between PND2 and 4 months (Figure 2A). Additional volcano plot analysis graphically displays the differential expression of 23,300 individual transcripts based on significance and fold change (Figure 2B). To determine functions associated with differential gene expression changes at each time point, Gene Ontology (GO) pathway analysis was performed. The bubble plot in Figure 3A visualizes the biological processes, cellular components, and molecular functions enriched by the differential data set and the table highlights the top 15 GO terms represented. These include biological processes such as cell proliferation, mitotic cell cycle, and defense response, along with cellular components such as extracellular matrix (ECM), indicating that valve maturation involves considerable changes in cell proliferation, ECM composition, and immune system programs. This is further highlighted in Figure 3B circle plot displaying genes which are known to be expressed in the heart valves based on previous publications, and their association with each GO term. More specific trends in these GO terms are shown in Figure 3C, with individually upregulated and downregulated genes in each category shown as red and blue dots, respectively. The inner rectangles are sized to positively correlate with the significance of each GO term, and colored to represent the overall direction of change in expression of each individual term. For example, the term “mitotic cell cycle process” has an overall down regulation at 4 months of age, while the “defense response” has an overall upregulation. In contrast, the “extracellular matrix” GO term is overall neither up-, nor downregulated, but the change in many individual transcripts is significant. Together these genomic analyses have defined transcriptional profiles of PND2 and 4 month aortic valve structures, and identified changes in functions associated with these mRNA patterns.

### Proliferation Programs Are Downregulated in 4 month Old Aortic Valves

**Figure 4:**
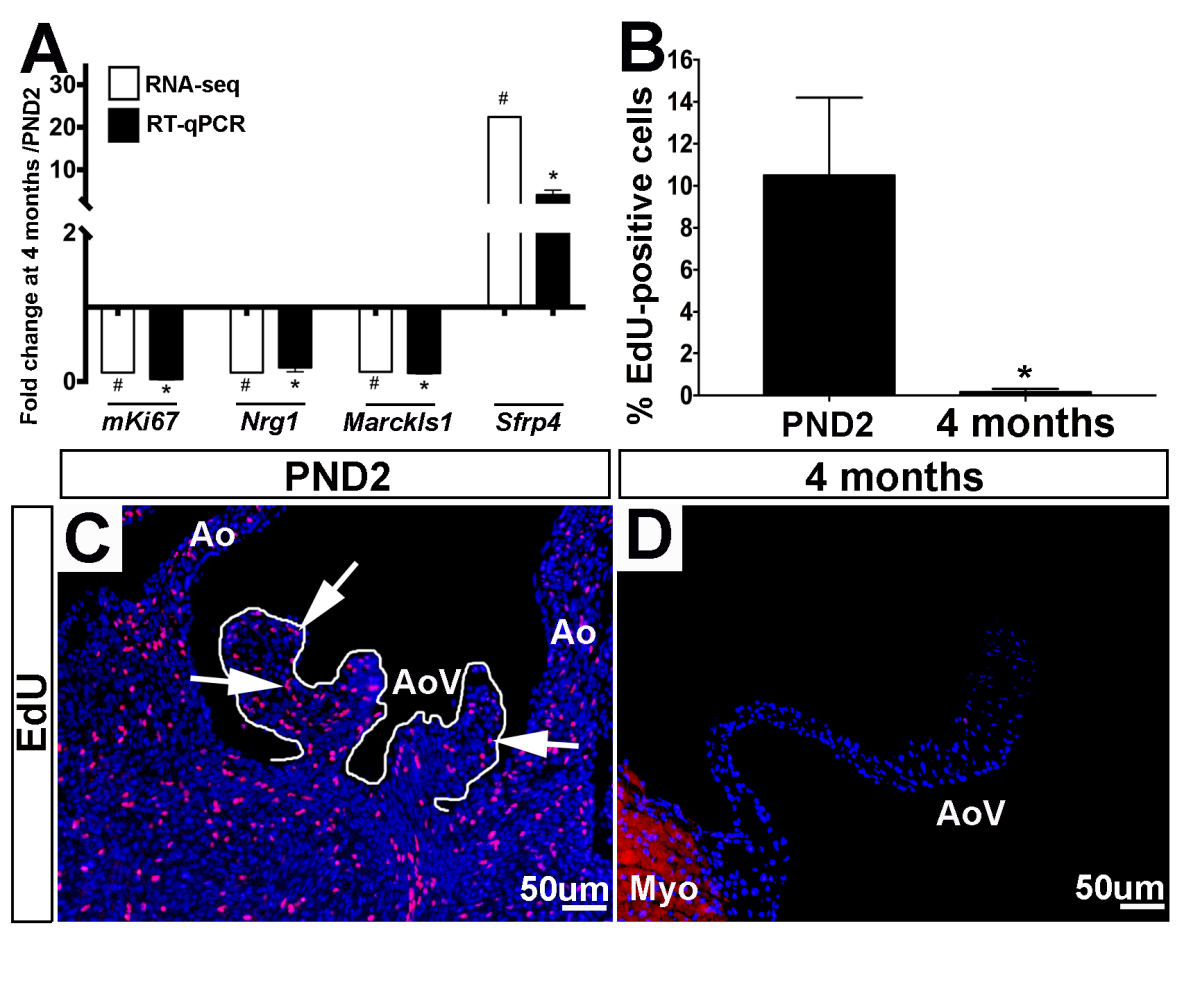
Positive regulators of cell proliferation are enriched at PND2 in murine aortic heart valves. **(A)** RT-qPCR performed on independent samples (black bars) validates the RNA-seq fold change (white bars) trends for proliferation-related genes. Expression is normalized to 1, indicated by the x-axis. (n=3, *:*p*<0.05; two-tailed unpaired *t*-test, #:FDR<0.05). **(B)** Quantification of 5-ethynyl-2′-deoxyuridine (EdU) positive cells as a percentage of total cell nuclei (indicated by DAPI, blue) at PND2 and 4 month time points. (n=3, *:*p*<0.05, two-tailed unpaired *t*-test). **(C)** Representative images of staining for incorporation of EdU into replicating DNA (white arrows point to EdU-positive cells) reveals high levels of proliferation in PND2 AoV as compared to **(D)** EdU staining in 4 month old aortic valve. Ao, aorta; AoV, aortic valve; Myo, myocardium.

Based on enrichment of cell proliferation-related genes from GO analysis (Table 2), we first validated the fold change trends observed by RNA-seq using RT-qPCR (Figure 4A) on independent biological samples. These validated genes include three positive regulators of proliferation *mKi67, Nrg1* and *Marcksl1*, which all decreased in 4 month old aortic valves, and an anti-proliferative gene, *Sfrp4*, found to have increased expression at this time point (Figure 4A) (25–28). To further validate mRNA findings, we utilized 5-ethynyl-2′-deoxyuridine (EdU) to visualize and compare the number of cells actively undergoing mitosis in the aortic valve at PND2 and 4 months of age (Figure 4B-D). At the earlier time point, ~10.5% of cells were found to be EdU-positive, while only 0.16% of cells were proliferating at 4 months (Figure 4B). These data are consistent with transcriptional changes and previous reports from our lab showing that ~6.2% of VECs (of the total VEC population) and ~3.3% of VICs (of the total VIC population) are proliferative at post natal stages, while in the young adult, proliferation rates of both VECs (~2%) and VICs (~1.1%) were significantly lower (4). Together, these observations suggest that a decline in cell division at 4 months is due to a combination of decreasing expression of postnatal proliferation programs while simultaneously increasing adult programs which inhibit proliferation.

**Table 2:** Genes included in the “Cell Proliferation” GO term.

**Downregulated at 4 months**	**Upregulated at 4 months**
AGTR2	ADIPOQ
H19	HSPA1A
BMP7	CD74
VASH2	PLA2G2D
IGF2	RBP4
IGF2BP1	H2-AA
FAM83D	H2-AB1
CTHRC1	VSIG4
CRH	CCL19
CDK1	CRLF1
NRK	CCL11
BEX1	NOX4
BUB1	SFRP4
IL31RA	CD209A
MELK	PTGFR
SCUBE2	BCL6
CCNB1	ITGAX
FIGNL1	KCNA1
AURKB	LGI4
UHRF1	ATF3
AGER	PTGS2
CDCA7L	AR
SHCBP1	ESR1
WISP1	CFB
NRG1	CEBPA
MKI67	APOD
HELLS	MMP12
ASPM	IL2RA
MCM10	RUNX3
IQGAP3	NR4A1
KIF20B	HSF4
MARCKSL1	CD274
BIRC5	ALDH3A1
RACGAP1	IFIT3
ROR2	FGF7
CHEK1	IL7R
TNC	CLEC11A
CDH3	WFDC1
SFRP2	AGAP2
E2F8	FLT3L
E2F7	FOLR2
SOX4	LIMS2
CDC20	GAPT
CENPF	SERPINF1
PTN	NR1D1
WNT4	FGF16
IRF6	BMPR1B
F2RL1	SERPINE2
FOXM1	IGHD
HMGA2	F3
MYCN	WNT10B
CD40	TRPV2
CDC6	CD28
MMP14	MLXIPL
TACC3	SIX1
EDN1	CPEB1
HMGB2	CYR61
SMARCA1	PTGIR
LAMC2	ECM1
GPC3	THPO
PROX1	CX3CL1
VASH1	ITGB2
CD276	ABCB1A
RNASEH2B	THRB
NASP	H2-M3
BLM	TOB2
ORC1	CHD5
NKX2-5	MUSTN1
CDK2	SAMD9L
TFRC	FTH1
LOXL2	PODN
CXADR	PTPRC
CASP3	FCGR2B
SLIT2	S100B
CAV3	FOSL2
DBN1	ATF5
EFNB2	COL18A1
FN1	BTG2
CDH5	PTPRU
SMYD2	COL4A3
TNFRSF13C	IL15
CDKN1C	NOV
DOT1L	FBLN1
RPS6KA2	JUN
SNAI2	PTAFR
MEG3	ADRB2
RIAN	SPN
RRM1	HAVCR2
HAS2	IL33
GM13275	RORA
DDR1	LRP1
ATPIF1	NTN1
GATA6	ITGAM
ERBB2	LEFTY1
MCM7	PID1
DISC1	GAS6
SCARB1	CST3
CTPS	EGR3
PTPRK	NAMPT
PTPRF	PDGFD
EDNRA	CD37
CD24A	CD9
SLC25A5	SLC11A1
TEK	CNN1
TRIM35	MVP
CNOT6	ANGPT1
KDM5B	H2-T23
PICALM	DOCK8
	TSPO
	CD86
	NACC2
	SPHK2
	HCLS1
	IRS2
	ZFP36
	CDKN1A
	TRF
	SLFN1
	IGFBP4
	RHBDD1
	DPT
	TNS2
	DOCK2
	NCF1
	PIK3CB
	APOE
	PAWR
	CD46
	CSF1
	IRF1
	CNTFR
	TACC2
	TGFBR3
	NUPR1
	BCL2
	VIPR2
	PTTG1
	TLR4
	RUNX2
	HYAL1
	IGFBP5
	CSF1R
	NCOA3
	PAK1
	PDE5A
	BRIP1
	CORO1A
	IFITM3

Genes are sorted according to fold change with the highest fold change at the top of the list.

### Postnatal and Adult Aortic Valves Have Distinct Extracellular Matrix mRNA Programs

**Figure 5:**
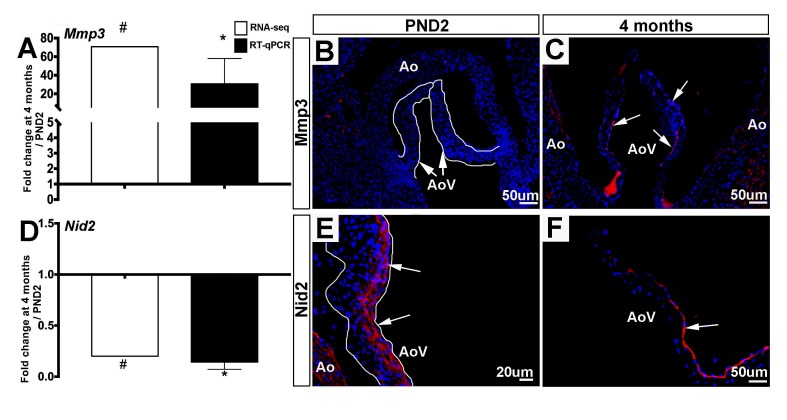
ECM molecular profiles between PND2 and 4 month old murine aortic valves are distinct.** (A)** RT-qPCR performed on independent samples (black bars) validates the trends seen in RNA-seq data (white bars). Expression is normalized to 1, indicated by the x-axis (n=3, *:*p*<0.05; two-tailed unpaired *t*-test, #:FDR<0.05). **(B)** Immunofluorescence staining for Mmp3 reveals low expression at PND2 compared to **(C)** 4 months (white arrows indicate Mmp3-positive cells). **(D)** RT-qPCR performed on independent samples (black bars) validates the trends seen in RNA-seq data (white bars) for ECM gene *Nid2*. Expression is normalized to 1, indicated by the x-axis (n=3, *:*p*<0.05; two-tailed unpaired *t*-test, #:FDR<0.05). **(E)** Immunofluorescence staining for Nid2 reveals high expression atPND2 (white arrows indicate Nid2-positive cells) compared to **(F)** Nid2 staining at the 4 month timepoint. Ao, aorta; AoV, aortic valve.

As indicated by the GO term z-score in Figure 3A, the overall expression of ECM transcripts does not significantly increase or decrease with age, yet there are considerable differences in the specific ECM-related mRNAs that are expressed between the two time points (Table 3). Matrix metalloproteinases, or Mmps, are known to be expressed in both healthy and diseased valves and are associated with physiological and pathological remodeling of the ECM respectively (29–31). Also known as stromelysin-1, Mmp3 targets degradation of proteoglycans, collagens, and elastins (32) and this Mmp family member has been described in cancer (33, 34), but little is known about Mmp3 in mouse valves. In this study, *Mmp3* increased from 1.22 reads per million kilobases (RPKM) at PND2 to 86.14 RPKM at 4 months. This significant increase was confirmed by RT-qPCR (Figure 5A,B) and immunofluorescence of aortic valve tissue sections, where Mmp3 is localized primarily to the sub-endothelial region of the leaflet (Figure 5B, Figure S1). In our previous VEC RNA-seq study, *Mmp3* transcript was not detected in the VEC population at any time point (4), and this is consistent with predominant expression of the protein in VICs; largely those close to the sub-endothelial location at 4 months (arrows, Figure 5C, Figure S1). In this study we show that the basement membrane ECM protein *Nidogen 2* (*Nid2*) was more highly detected in PND2 samples at 76 RPKM, while only 15 RPKM were detected at 4 months. This expression pattern was confirmed by RT-qPCR (Figure 5D) and immunofluorescence data shows broad Nid2 localization in both VECs and VICs at PND2, but more localized within the endothelial cell layer at 4 months (Figure 5E,F, Figure S1), and this pattern of decreased expression with maturation is consistent at the RNA level in VECs as previously described (4). These data show that PND2 and 4 month old aortic valves have distinct ECM-related transcriptional profiles associated with growth and maintenance respectively.

**Table 3:** Genes included in the “Extracellular Matrix” GO term.

**Downregulated at 4 months**	**Upregulated at 4 months**
S100A9	MYOC
COL24A1	MMP3
BMP7	GLDN
2010005H15RIK	CHAD
CTHRC1	MMP10
FREM2	COMP
COL26A1	ENTPD2
ELN	LAMC3
COL9A1	CILP2
FREM1	SOD3
ADAMTS12	FBLN7
FRAS1	NPNT
MFAP2	PRELP
ADAMTS17	MMP12
WISP1	COL4A6
HMCN1	VIT
LAMA1	CCBE1
COL12A1	CPXM2
ACAN	OPTC
TNC	SERPINF1
FREM3	SERPINE2
LAMB1	F3
LOX	CRISPLD2
EMILIN3	WNT10B
TFPI2	EPYC
NID2	AEBP1
PTN	CYR61
WNT4	ECM1
MMP15	ECM2
FBLN2	PODN
MMP14	SMOC2
TPSB2	COL5A3
PXDN	TIMP3
LAMC2	SMOC1
GPC3	COL18A1
ADAMTS9	DCN
GPC2	HIST1H4C
NID1	COL4A3
LOXL2	NOV
ANGPTL4	FBLN1
SLIT2	SPARCL1
WNT5B	SPN
VCAN	NTN1
CMA1	RARRES2
FN1	CST3
COL5A1	LGALS3BP
COL5A2	IGFBP7
LAMA3	VWA1
P3H1	MGP
TUBB5	TNXB
AGRN	COL15A1
SFPQ	BGN
ITGA6	THSD4
LAMC1	HIST1H4A
SLC25A5	TRF
CD93	LAMB2
COL4A1	DPT
	LMCD1
	APOE
	ADAMTSL5
	TGFBR3

Genes are sorted according to fold change with the highest fold change at the top of the list.

### Gene Ontology Defense Response Markers Are Highly Enriched in 4 month Old Aortic Valves

**Figure 6:**
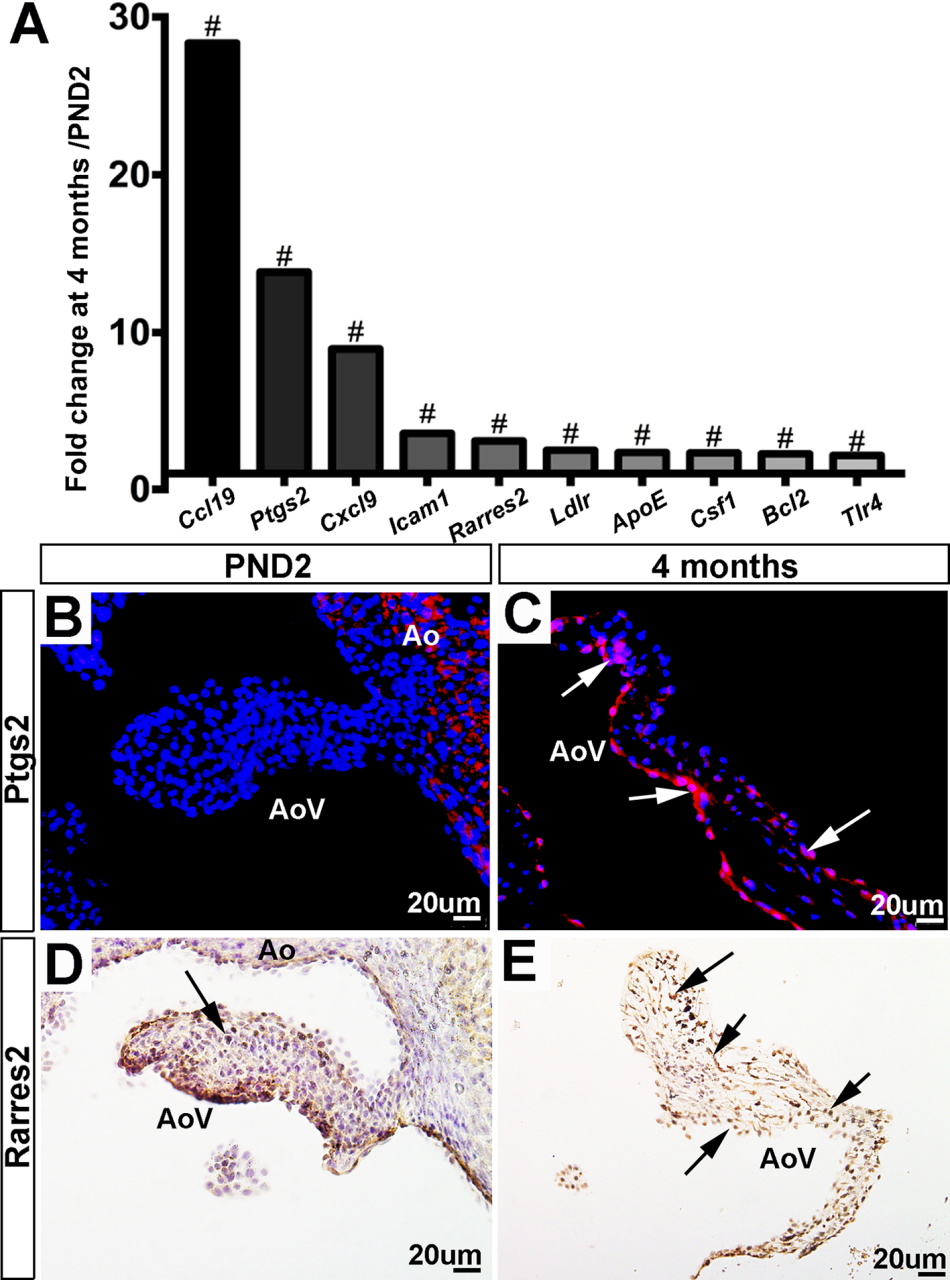
Expression of defense-related genes increases with valve maturation. **(A)** RNA-sequencing fold changes of defense-related genes. Expression is normalized to 1, indicated by the x-axis (#:FDR < 0.05). **(B)** Immunofluorescence staining for Ptgs2 validates RNA-seq trends, showing low expression at PND2 compared to **(C)** Ptgs2 staining at the 4 month time point (white arrows indicate Ptgs2-positive cells). **(D)** Immunofluorescence staining for Rarres2 validates RNA-seq trends, showing low expression at PND2 compared to **(E)** Rarres2 staining at the 4 month time point. Ao, aorta; AoV, aortic valve.

As shown in Figure 6A and Table 4, a large majority (77%) of defense response genes are most highly expressed in aortic valve structures at 4 months of age and include *Ccl19, Ptgs2* and *Cxcl9*. The increased expression of *Ptgs2* (also known as *Cox2*) (Figure 6B,C) and *Rarres2* (also known as *﻿Tig2*) (Figure 6D,E) are confirmed here by immunofluorescence. Ptgs2 is an enzyme involved in the synthesis of prostaglandins which are known to mediate pain and inflammation responses (35), and has been described in the valve as a pro-osteogenic marker (36). Consistent with this previous valve study, we observed expression towards the endothelium at 4 months of age (Figure 6C, Figure S1), and this is consistent with RNA expression in the VEC population (4). Like Ptgs2, Rarres2 is also known to regulate inflammation and has been linked with hypertension (37), a known risk factor of aortic valve stenosis (38). By immunofluorescence, Rarres2 is widely expressed throughout the valve leaflet including VECs and VICs at 4 months of age, which is a significant increase compared to PND2 (4). Overall, our RNA-seq data shows that expression of defense response-related genes increases with age in the murine aortic valve.

**Table 4:** Genes included in the “Defense Response” GO term.

**Downregulated at 4 months**	**Upregulated at 4 months**
AGTR2	CFD
S100A9	ADIPOQ
CITED1	HAMP
IGF2	HP
COLEC10	CD74
NGP	CCL8
C1QTNF3	H2-AA
CRH	H2-EB1
S100A8	H2-AB1
IL31RA	CLEC10A
ADAMTS12	GM2564
CHAF1B	CCL19
AGER	CCL11
PBK	C4B
ULBP1	C4A
RNASEL	PTGFR
RAET1E	BCL6
ELF3	ITGAX
BRINP1	PTGS2
RAET1D	ESR1
F2RL1	CFB
CD40	APOD
LCK	IL2RA
MASP1	H2-Q7
TPSB2	FFAR4
MDK	CXCL9
EDN1	ITIH4
HMGB2	SLAMF7
SLFN9	C1S1
CPA3	IFIT3
CD276	C1S2
TSPAN6	GBP2
TYRO3	FGF7
CASP6	C1RA
CCR1	C1RB
SLIT2	WFDC1
FN1	OAS2
GM13275	GPR17
GGT5	PELI3
HYAL2	MYLK
EDNRA	CFH
CD24A	ISG20
FANCA	H2-K1
TRIM35	SERPINF1
SUSD4	NR1D1
CD93	CLEC7A
SLC35B3	BMPR1B
	C1RL
	F3
	LYZ1
	TGTP1
	SLAMF8
	TGTP2
	ITK
	HRH1
	ALOX5
	CD28
	NR1H3
	PSTPIP1
	IRAK3
	LYZ2
	H2-D1
	TAP2
	APOBEC1
	GNG7
	PTGIR
	ECM1
	CX3CL1
	ITGB2
	CD14
	CCRL2
	HIST2H3C2
	GBP6
	GBP10
	H2-M3
	C3
	MILL2
	SERPING1
	CYBB
	PTPRC
	FCGR2B
	RAB7B
	HIST1H2BE
	TRIM30A
	NFKBIZ
	ICAM1
	SLFN8
	IFIH1
	HIST1H2BJ
	FAS
	IL15
	NOV
	PTAFR
	MAP1A
	CASP1
	MYO1F
	ADRB2
	FGF14
	HIST1H2BC
	SPN
	HAVCR2
	IL33
	B2M
	COLEC12
	RORA
	ITGAM
	TAP1
	RARRES2
	CST3
	ZC3H12A
	IRGM2
	NAIP5
	HIST1H2BL
	IGTP
	PNMA1
	HIST1H2BK
	CD37
	TLR8
	SLC11A1
	MGLL
	IL17RE
	HIST1H2BN
	CADM1
	H2-T23
	HERC6
	CD86
	SETD6
	ZFP36
	THEMIS2
	LDLR
	HFE
	C1QA
	IGFBP4
	LMCD1
	ALOX5AP
	NCF1
	GBP8
	APOE
	TNFRSF25
	SERPINB9
	CD46
	CSF1
	IRF1
	NUPR1
	BCL2
	STAR
	DRD1
	PIK3AP1
	IRF8
	TLR4
	TLR3
	BIRC3
	HYAL1
	CSF1R
	C1QB
	UNC93B1
	TRIM21
	PDE5A
	PTGIS
	CORO1A
	AOAH
	IFITM3

Genes are sorted according to fold change with the highest fold change at the top of the list.

## Discussion

This current study explores transcriptional differences in PND2 and 4 month old murine aortic valve expression profiles with the goal of identifying genetic programs representative of valve growth and valve maintenance, respectively. Long term, this may be important for the development of alternative therapies; specifically, those exploring the growth or regenerative capacity of adult diseased valves. Our results indicate that at PND2, dynamic leaflet growth is associated with a unique transcriptional profile compared to homeostatic adult valves at 4 months. Of the 23,303 detectable genes in our RNA-seq data, the number of differentially expressed transcripts found to be up and down regulated at each time point were approximately equal, suggesting that gene transcription patterns were not overtly altered, but rather transitioned from a postnatal to adult expression profile. Such profiles are related to enriched biological functions including cell proliferation at PND2 and defense response at 4 months of age. Interestingly, ECM components were enriched at both time points, but the associated mRNA profiles were unique. Together these analyses contribute to the current knowledge and further advance our understanding of the molecular signatures and biological functions characteristic of the whole aortic valve structure at PND2 and 4 months. provide critical information related to genetic programs in the growing and homeostatic heart valve.

PND2 murine aortic valves are defined by a specific transcriptional profile including the unique expression of 602 genes (Figure 1C, Table 1) that were not detected at 4 months of age. In addition, 1,801 transcripts were upregulated at PND2, while 1,858 were decreased compared to the adult homeostatic valve. According to Gene Ontology analysis, many of the transcripts enriched at PND2 suggest an overall upregulation of active cell proliferation by a specific set of genes. These include increased expression of those associated with active cell division (*Ki67, Aurkb*), pro-proliferation markers (*Bub1, Cdk1, Foxm1, Nrg1*) (27, 39, 40) and decreased expression of proliferation inhibitors (*Nox4, Sfrp*) (41–44). This specific expression profile of cell proliferation markers supports EdU observations and represents active elongation of the immature valve structure. It will become important to understand how this molecular signature of cell proliferation is downregulated after maturation is complete and explore the potential of re-introducing key regulators to stimulate cell division and replenish dysfunctional cell populations in the adult following injury or disease.

In addition to high levels of cell proliferation, the PND2 valve is characterized by a specific ECM mRNA profile (Table 3) which likely corresponds to the mechanical demands during the postnatal period. When comparing PND2 to 4 months, RNA-seq analysis reveals significant enrichment of highly expressed fibrillar collagens including *Col24a1*, *Col9a1*, and *Col5a1*, indicating the need for additional stability as the growing postnatal valve adapts to hemodynamic changes in response to closing of the foramen ovale (45, 46). RNA-seq analysis also uncovered higher PND2 levels of ECM proteins such as *Frem1/Frem2* and *Nidogen2* (*Nid2*), which have been shown to stabilize basement membranes underlying endothelial cells and may provide further structural integrity to the developing valve (47, 48). Besides identifying the enrichment of differentially expressed fibrillary collagens and specific proteoglycans, RNA-seq analysis shows that the PND2 valve also expresses a distinct profile of ECM enzymes, such as *Mmp15* and *Adamts17*, indicating the need for physiological remodeling during growth and maturation

The 4 month adult valve is physically and molecularly distinct from the postnatal valve, with elongated leaflets containing distinct layers of collagen, proteoglycan, and elastin (Figure 1B). At this time point, 477 transcripts were found to be uniquely expressed, with the most abundant unique mRNAs including *Cfd*, *Retnla*, and *Clec3a*. In contrast to the PND2 aortic valve, the adult valve displays significantly decreased levels of cell proliferation, likely due to increased expression of proliferation inhibitors and lack of enrichment of positive regulators. At 4 months, the valve ECM is diverse compared to PND2 (Table 3) and likely reflects differences in biomechanical demand in response to the adult circulatory system (49, 50). Similar to the PND2 valve, collagens and proteoglycans are predominant. However, the most highly differentially expressed collagens are those associated with basement membranes, including *Col4a6* and *Col18a1*, which act as a cell scaffold to maintain current cell populations and cell integrity as opposed to providing support for high cell turnover. In addition, the proteoglycan profile is moved towards enrichment of decorin (*DCN*) and biglycan (*BGN*) consistent with previous studies in aging pigs (51). Furthermore, the contribution of ECM remodeling enzymes is shifted to *Mmp3* and its inhibitor *Timp3* at 4 months, possibly indicating a differential need of the ECM to sustain homeostasis. Previous studies have suggested correlations between VIC phenotype and ECM composition (52, 53) and therefore we anticipate that our findings at 4 months are related to the quiescent VICs, while the diversity at PND2 is dictated by proliferative and active VICs.

One of the most prominent differences in expression profiles between the PND2 and 4 month aortic valve is the considerable upregulation of defense-related transcripts at the older time point, indicating increased immune system activation with valve maturation (Figure 6, Table 4). Previous studies from other groups have shown that the appearance of immune markers such as *Ptgs2* and *Rarres2* precedes the onset of disease both in the heart valve and other cardiovascular systems (36, 37). In addition, there is increasing evidence to suggest that inflammation in the valve is an initial homeostatic repair mechanism activated in response to minor valve injuries sustained throughout life, but that this repair mechanism may become pathogenic if overactive or long-lasting [reviewed (54)]. Our study suggests that some level of activation of the immune system in the valve is present at 4 months of age under homeostatic conditions, however, it is not clear whether these defense markers are an early indication of valve degeneration, or a root cause of disease themselves. Further investigation into target genes such as *Cfd* and *Adipoq* will give insight into the possible role of defense response genes in valve disease therapeutics.

Our data shows that postnatal heart valves contain highly proliferative VICs producing a distinctive set of postnatal ECM proteins, while adult VICs are mainly quiescent and are associated with a very different ECM composition as well as increased defense response markers. A limitation of our study is that RNA-seq analysis was performed on whole valve tissue and therefore RNA profiles cannot be distinguished between VEC and VIC populations, however IHC studies shown here, in combination with a previous study from our group (4) support enrichment towards one cell type. Nonetheless, we have unveiled multiple conceivable processes that contribute to postnatal valve maturation and maintainence that pave the way for elucidiating mechanisms underlying valve defects present at birth and those acquired later in life. Furthermore, the basic priciples of cell proliferation and ECM remodeling may also be applied to valvulogenesis and congenital valve malformation. From here further research is needed to determine how findings in this study can be used to develop alternative therapeutic strategies to promote self-repair, replenishment and regeneration of valve cell populations in pathogenesis.

## Ethics Statement

All animal procedures were approved by The Research Institute at Nationwide Children’s Hospital Institutional Animal Care and Use Committee (Protocol # AR13-00054).

## Author Contributions

The experimental data was collected by EN, and RNA-seq performed and analyzed by CN. Additional analysis of RNA-seq data was undertaken by SL. EN and JL generated the manuscript with input from SL. The entire study was overseen by JL.

## Conflict of Interest Statement

The authors declare that the research was conducted in the absence of any commercial or financial relationships that could be construed as a potential conflict of interest.

## Supplementary material

Figure S1 Cell localization of Mmp3, Nid2 and Ptgs2 in the aortic valve at 4 months of age. Immunohistochemistry to show Mmp3 **(A)**, Nid2 **(B)** and Ptgs2 **(C)** expression (red) relative to CD31 (endothelial cells, green) in 4 month old aortic valve leaflets. Arrows indicate protein expression in VICs, arrowheads denote staining in CD31-positive VECs.
