# Corrigendum: Postnatal and Adult Aortic Heart Valves Have Distinctive Transcriptional Profiles Associated With Valve Tissue Growth and Maintenance Respectively

**DOI:** 10.3389/fcvm.2019.00164

**Published:** 2019-11-15

**Authors:** Emily Nordquist, Stephanie LaHaye, Casey Nagel, Joy Lincoln

**Affiliations:** ^1^Molecular Cellular and Developmental Biology Graduate Program, The Ohio State University, Columbus, OH, United States; ^2^Center for Cardiovascular Research, The Research Institute at Nationwide Children's Hospital, Columbus, OH, United States; ^3^The Heart Center, Nationwide Children's Hospital, Columbus, OH, United States; ^4^The Institute for Genomic Medicine at Nationwide Children's Hospital, Columbus, OH, United States; ^5^Ocean Ridge Biosciences, Deerfield Beach, FL, United States; ^6^Department of Pediatrics, The Ohio State University, Columbus, OH, United States

**Keywords:** aortic valve, RNA-sequencing, mRNA, cell proliferation, extracellular matrix, postnatal, adult

In the original article, there was a mistake in the table listing the **qRT-PCR** primers, in the **Materials and Methods** as published. The forward and reverse primers were duplicated for Mki67. The corrected **qRT-PCR** primer table appears below.

The authors apologize for this error and state that this does not change the scientific conclusions of the article in any way. The original article has been updated.

**Table:** 

**Gene name**	**Forward primer sequence (5^**′**^ to 3^**′**^)**	**Reverse primer sequence (5^**′**^ to 3^**′**^)**
***Nid2***	AGGAGTGAGCATGTTTCGG	AGGGGTATTGCCAGCTTCAC
***Mmp3***	TGCATGACAGTGCAAGGGAT	ACACCACACCTGGGCTTATG
***Marckls1***	CCCGTGAACGGAACAGATGA	CCCACCCTCCTTCCGATTTC
***Gsn***	GGGACGGCCGGTTACTTAAA	CTTCAGGAATTCGGGGTGCT
***Filip1l***	AGGCTCCACTGCTGGATTTC	GACTTCTCTGACACGGGACG
***Myoc***	ACGACACTAAAACGGGGACC	TTCTGGCCTTTGCTGGTAGG
***Retnla***	GGAACTTCTTGCCAATCCAGC	CAGTGGTCCAGTCAACGAGT
***Npdc***	GCACTCCCGACACTTTTCTC	GGTACCCACTCCGGGAACT
***Sfrp4***	CCTGGCAACATACCTGAGCA	AGCATCATCCTTGAACGCCA
***Mki67***	AGAGCTAACTTGCGCTGACT	ACTCCTTCCAAACAGGCAGG
***Nrg1***	CCATCTCTCGATGGGCTTCC	ATGCAGAGGCAGAGGCTTAC
***Nrep***	GCATGATGCCCTTTTTCATCCA	TCCTTAGGCACGGGAAGTCT
***Acta2***	CCTTCGTGACTACTGCCGAG	GAAGGTAGACAGCGAAGCCA
***Dlk1***	AGAGTACCCCTCTCCTCACC	CGCCGCTGTTATACTGCAAC
***Cfd***	TACATGGCTTCCGTGCAAGT	GGGTGAGGCACTACACTCTG

