# Supplementary figures and images for: Postnatal and Adult Aortic Heart Valves Have Distinctive Transcriptional Profiles Associated With Valve Tissue Growth and Maintenance Respectively
Source: Front Cardiovasc Med. 2018 Apr 24;5:30. doi: 10.3389/fcvm.2018.00030 (PMC5928323; doi:10.3389/fcvm.2018.00030)

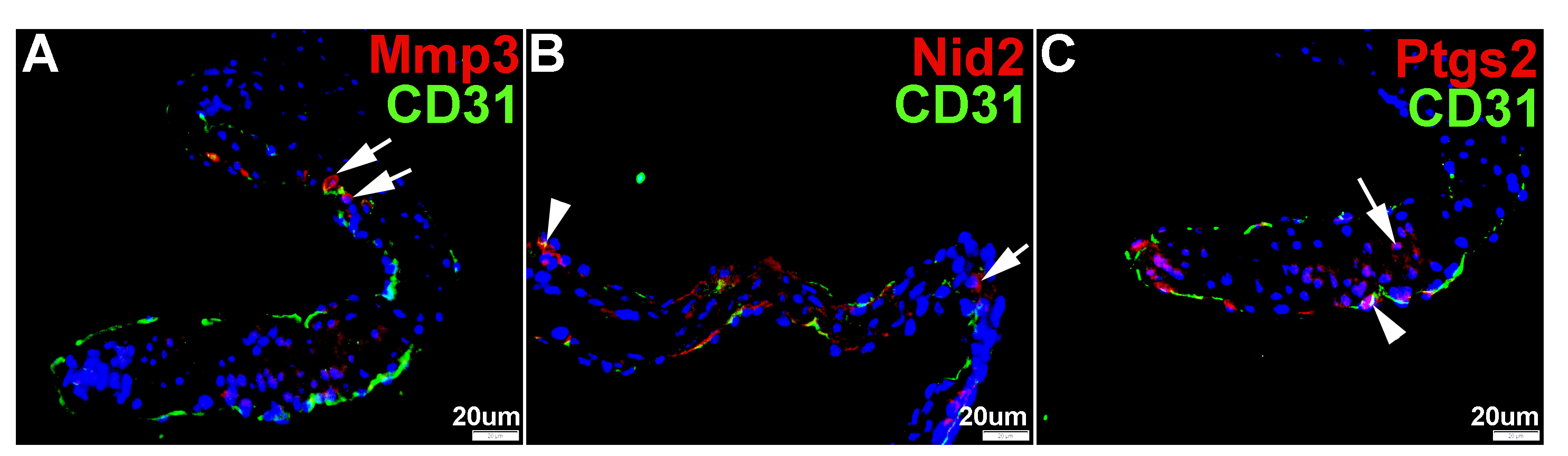

Supplement: Figure S1 — Cell localization of Mmp3, Nid2 and Ptgs2 in the aortic valve at 4 months of age. Immunohistochemistry to show Mmp3 (A), Nid2 (B) and Ptgs2 (C) expression (red) relative to CD31 (endothelial cells, green) in 4 month old aortic valve leaflets. Arrows indicate protein expression in VICs, arrowheads denote staining in CD31-positive VECs. [file Image1.tif]
